# Assessment of patient safety culture in Moroccan primary health care: a multicentric study

**DOI:** 10.1186/s12912-024-01864-6

**Published:** 2024-03-21

**Authors:** Chaima Fihri Fassi, Yasmine Mourajid, David Mawufemor Azilagbetor, Asma Sabri, Mohamed Chahboune, Abderraouf Hilali

**Affiliations:** 1 Higher Institute of Health Sciences, Laboratory of Health Sciences and Technologies, Hassan First University of Settat, Settat, Morocco; 2 University of Basel, Basel, Switzerland

**Keywords:** Patient safety, Patient safety culture, Primary healthcare settings, Morocco

## Abstract

**Background:**

Promoting patient safety is a critical concern for developing-countries health systems like Morocco. There is an increasing acknowledgment of the need to create a patient-centered culture with the aim to decrease the number of adverse events related to care and improve health-care quality in Morocco.

**Objective:**

The purpose of this study is to examine the perceptions of health professionals working in primary care level of care facilities in Morocco about the concept of patient safety culture.

**Methods:**

We conducted a multicentric cross-sectional study of a quantitative nature in primary healthcare facilities in ten Moroccan cities, measuring ten patient safety culture dimensions, from February 2022 to June 2022. Data was collected using the French version of the HSOPSC questionnaire.

**Results:**

The most developed dimension of the culture of patient safety was found to be Teamwork within Units (69%), followed by Supervisor/Manager’s Expectations & Actions Promoting Patient Safety (59%). The least developed dimensions were Staffing (34%) and Nonpunitive Response to Errors (37%).

**Conclusion:**

Improving patient safety culture should be a priority for primary healthcare facility administrators and all stakeholders, addressing, in particular, the shortage of human resources. In addition, health personnel should be encouraged to report errors without fear of punitive consequences.

**Supplementary Information:**

The online version contains supplementary material available at 10.1186/s12912-024-01864-6.

## Introduction

Patient safety is a critical element of quality healthcare due to the fact that it involves minimizing and preventing adverse events that patients are exposed to in healthcare institutions. Worldwide, there is still a major challenge with unsafe healthcare [1]. Although there has been a notable improvement in patient safety over the past ten years, there are still many gaps, and the harm caused to patients by adverse healthcare events is still important [2]. Therefore, the emphasis on enhancing patient safety culture as a tool to monitor the development of patient safety measures and cultural transition has grown through time [3]. There are several definitions of safety culture but the most common universal definition is “the shared attitudes, beliefs, values, and assumptions that drive how people see patterns of behavior in safety performance inside their organization” [4]. Consequently, building a patient safety culture among healthcare professionals entails an awareness of what is important in a healthcare organization and what attitudes and actions linked to patient safety are expected and suitable [5, 6].

The WHO stated in 2018 that patient safety in primary care is a concern that necessitates local and permanent solutions, and that assessing the patient safety culture should be one of the initial measures [7]. Measuring a patient safety culture, particularly from the perspective of health professionals, allows for an assessment of strengths and places for advancement. It also allows for the development of suitable methods for evaluating new safety programs through comparisons of before and after results [8]. 

The present state of international research about patient safety culture in healthcare organizations in general, as well as the research methodologies used to assess PSC in hospitals settings [9] and primary care settings, are not sufficient. Primary care facilities are regarded as the entry point into the healthcare system, focused on treatment, disease prevention, and quality of life, regardless of the availability and continuity of care in primary care, research at this level is less common than in hospitals [10] Such limitations could obstruct present efforts to improve patient safety around the world. However, a significant percentage of medical consultations occur in primary care facilities, and numerous adverse events in health structures happen there, emphasizing the importance of primary care patient safety research [11]. There are no uniform criteria for identifying and classifying patient safety potential risks in primary care. According to the research, 24–85% of all major adverse events are preventable [12]. Hence constructing and promoting a patient safety culture among its professionals is a critical first step to reduce and prevent adverse events in primary care [13], it depends on the ability of an institution to build a patient safety culture that has the potential to be strengthened. Through dedication to discussing and learning from mistakes, acknowledgment of the inevitability of errors, proactive detection of latent risks, and implementation of a nonpunitive system for reporting and analyzing adverse events are the key components of promoting a safety culture among health professionals [14]. In addition, communication with in health organizations with established positive patient safety culture is characterized by trust among individuals and shared perceptions of the importance of patient safety and the efficiency of preventive measures [15]. 

Moroccan health-care system is organized into public and private health institutions. The public structures are divided into three divisions of care. Primary healthcare centers, offering primary and essential care to the general population, are a good example of forefront structures. The second level is provincial hospitals that refers to a division in the healthcare system in which patients from primary care become referred to doctors in higher-level structures for treatment and advanced diagnosis. The third level of care delivers specialized medical consulting care, frequently on reference from primary and secondary care, in addition to health research and academic training. Primary healthcare centers are the first level of contact for patients, thus quality and patient safety are critical [16].

With regard to the clear discrepancies in knowledge in Morocco concerning patient safety in primary care and the association between patient safety culture and safe care, this study centered on the issues that follow: In what way is the patient safety culture portrayed among the various groups of primary care professionals? Is there a difference in the patient safety culture perception among the primary care professionals? Hence, the purpose of this article was to assess the level and the perception of patient safety cultures among primary care professionals.

## Methodology

### Study setting and participants

In this research we executed a questionnaire of descriptive nature is carried out from February 2022 to June 2022. The study was conducted in primary healthcare centers (*n* = 350) in ten cities of Morocco (Rabat, Casablanca, Marrakech, Fez, Tangier, Tetouan, Laayoune, Meknes, Settat and Berrechid). We opted for a two-stage random sampling. Therefore, we selected ten provinces through a random draw, then chose a set number of urban health centers at random within each province’s capital city, and we exhaustively targeted all of the experts who work at these centers (*n* = 811) including doctors, nurses, midwives, and other staff members of primary healthcare centers in the 10 Moroccan cities mentioned above, were eligible to participate in the survey. However, new medical and nursing staff with less than one month of experience, as well as trainee medical and nursing staff, were excluded from this study. As it was advocated by the Coordination Committee of the Clinical Evaluation and Quality in Aquitaine (CCECQA) in the questionnaire’s user guide [17]. The participation in the survey was voluntary and anonymous for all the participants. The study’s major variable is the culture of patient safety characteristics as perceived by medical, nursing, and administrative employees in primary healthcare facilities. Other variables were socio-demographic information, including the participants’ profession, years of experience, percentage of time spent at work, and involvement in healthcare safety committees.

### Study instrument

The instrument of data collection considered in this study is the Hospital Survey on Patient Safety Culture (HSOPSC) questionnaire created under the management of the American Agency for Healthcare Research and Quality is the data gathering tool employed by this study. This tool has been applied in several countries including the Netherlands, the United States, Germany, Australia, Brazil, the United Kingdom, Turkey, Saudi Arabia and Kuwait. This research utilized the French translation and validation of the Hospital Survey on Patients Safety Culture (HSOPSC) questionnaire [18]. Given its favorable psychometric features, it is the most often used instrument for assessing PSC, it is an accredited and valid survey that accurately measures the research conception of (PSC) [19].

The French version of the questionnaire has been constructed up of ten PSC dimensions in addition to two separate questions asking respondents to give an overall rating of patient safety in their work units and to indicate the number of events they have reported in the last 12 months. Also, respondents are asked to provide their demographic information (their work unit, city, occupation, seniority in the profession as well as in the current establishment, membership in committees/structures of risk management, etc.) investigated through 45 items to assess the beliefs, skills, and behaviors associated with the safety culture of the institution from the perspective of the health professionals, organized according to the following structure:

D1: Overall perception of patient safety, D2: Frequency of events reported, D3: Supervisor/manager expectations and actions promoting patient safety, D4: Organizational Learning—Continuous Improvement, D5: Teamwork within units, D6: Communication openness, D7: Non-punitive response to error, D8: Staffing, D9: Management support for patient safety, D10: Teamwork across units, each dimension is composed of three to four items constructed in a positive or negative manner.

The participant may select a score on a five-point Likert scale for each question, with response options that vary from (5) strongly agreeing to (1) strongly disagreeing, Scores (4) agree and (5) strongly agree are thought of as ‘positive’ in regard to PSC, while scores (3) are considered ‘neutral’ and scores (1) strongly disagree and (2) disagree are considered ‘negative’ in relation to PSC. The percentage of positive answers for every dimension of the HSOPS was the primary result. Because an answer on a negatively worded item implies a positive response, it was reverse-coded.

Regarding the additional questions, the “patient safety grade,” the participant had responses options of excellent, good, very good, poor or failing, and the “number of events reported”, with response options of no events reported, 1 to 2 events reported, 3 to 5 events reported, 6 to 10 events reported, 11 or more events reported.

Data was collected in the primary healthcare facilities of the cities included in this research’s sample from March 02 to May 20, 2022. Prior to embarking on a large-scale data collection, a pre-test was conducted with ten health professionals in one primary center to ensure the clarity of the questionnaire. The entire research process was conducted in French because Morocco is a French-speaking country and our study instrument and guide are in French.

### Data analysis

Data were entered into a Ms Excel document, exported and analyzed by the IBM SPSS Statistics version 20 software. Questionnaires containing incomplete answers, or several answers for a single question were eliminated before data analysis. During the statistical analyses, the 16 negative items were re-coded: 5, 4, 3, 2 and 1 were attributed to *Totally disagree*, *Disagree*, *Neither*, *Agree* and *Strongly agree* respectively, as well as *Never*, *Rarely*, *Sometimes*, *Most of the time* and *always* respectively.

Three modalities of responses; *Positive*, *Neutral* and *Negative* were adopted for the five response options: Positive Responses (Agree + Strongly Agree / Most of the time + Always), Neutral (Neutral / Sometimes) and Negative Responses (Totally disagree + Disagree / Never + Rarely). The negative items were also adapted to these 3 modalities by means of a re-coding.

A general score in percentage (%) was calculated for each dimension by finding a general average of the Positive Responses of all the items found in a specific dimension composite. A dimension is said to be “Developed” or “Positive” if the general average of its items is at least 75%. On the contrary, a dimension is said to be “Negative” or “to be Improved” if the general average of its items is less than 50%.

## Results

### Characteristics of the participants

The demographic characteristics associated with survey respondents are shown first in the following sections. subsequently, we assessed the level of patient safety culture among healthcare personnel. In total, of 923 healthcare personnel who participated in the study, 857 completed the questionnaire. The response rates were 92.8%. After eliminating 46 responses that were either not answered completely or the respondent selected more than one answer for the same question, we included 811 responses in the data analysis. The responses, classified according to their respective cities are as follows: Rabat (*n* = 72), Casablanca (*n* = 188), Marrakech (*n* = 119), Fez (*n* = 110), Tangier (*n* = 120), Tetouan (*n* = 19), Laayoune (*n* = 54), Meknes (*n* = 70), Settat (*n* = 39) and Berrechid (*n* = 20). Therefor 53% of respondents had more than ten years of experience at the hospital. nurses represent more than half of the sample (57.3%), midwives 22.3%, doctors 19.1%, and dentists, an ophthalmologist and physiotherapists represent 1.2%. The demographic characteristics of the final sample are described in Table 1.

**Table 1:** Characteristics of participants

SOCIO-DEMOGRAPHIC CHARACTERISTICS	*n* = 811	%
PROFESSION		
Physician	155	19.1
Nurse	465	57.3
Midwife	181	22.3
Dentist	7	0.9
Ophthalmologist	1	0.1
Physiotherapist	2	0.2
PROFESSIONAL WORK EXPERIENCE		
Less than 1 year	74	9.1
1 to 2 years	72	8.9
3 to 5 years	91	11.2
6 to 10 years	139	17.1
11 years or more	435	53.6
WORK EXPERIENCE IN THE CURRENT ESTABLISHMENT		
Less than 1 year	183	22.6
1 to 2 years	127	15.7
3 to 5 years	160	19.7
6 years or more	340	41.9
WORKLOAD		
Work occupies less than 50% of working time	85	10.5
Work occupies more than 50% of working time	725	89.4
PARTICIPATION IN RISK MANAGEMENT COMMITTEES OR STRUCTURES		
Yes	130	16.0
No	681	84.0

### PSC and adverse events reports

The overall assessment of patient safety culture among healthcare professionals was rated as acceptable in 41% of cases and poor in 19% of them. The frequency of reported adverse events scored at (67%) as the total professionals said they did not report any AE in the previous 12 months as described in Table 2.

**Table 2:** Level of care safety and number of adverse events reported in the past 12 months

LEVEL OF CARE SAFETY AND NUMBER OF ADVERSE EVENTS REPORTED IN THE PAST 12 MONTHS			
Item		*n* = 811	%
The level of care safety	Excellent	51	6.3
	Very good	245	30.2
	Acceptable	334	41.2
	Poor	156	19.2
	Failing	25	3.1
		*n* = 811	%
The number of adverse events reported	No forms filled (event reports)	540	66.6
	1 to 2 events reports	181	22.3
	3 to 5 events reports	42	5.2
	6 to 10 events reports	29	3.6
	11 to 20 events reports	18	2.2
	More than 20 events reports	1	0.1

### Patients safety culture dimensions

The overall perception of patient safety had an average positive score of 56%. Five dimensions had scores between 50% and 70% they are all underdeveloped and five dimensions that were undeveloped with less than 50% scores.

The percentage of positive comments for teamwork within units was highest (69%). Professionals perceived that the individuals supported one another, collaborated as a team, and treated one another with decency. In addition, they had a belief that by communicating with their colleagues, they improved their safety care practice. The lowest scores were attributed to the following dimensions:

- D8 (Staffing (34%)): professionals believed there were not enough people to undertake the workload. Furthermore, they had the impression that they were always functioning in an emergency mode.
- D7 (Non-punitive response to error (37%)): personnel focused on the concern of assigning blame for an error to a particular person.
- D10 (Teamwork across units (40%)): the personnel identified dysfunctions during inter-departmental exchanges and communication.

The description of the final results of all PSC dimensions and items are shown in (Table 3).

**Table 3:** Scores and items of the 10 dimensions of safety culture

Items of safety culture dimensions at the primary health care centers	Positive responses (%)
**D1: Overall perceptions of safety**	56
Patient safety is never sacrificed to get more work done	69
Our procedures and systems are good at preventing errors from happening	62
It is just by chance that more serious mistakes do not happen around here	46
We have patient safety problems in this facility	47
**D2: Frequency of events reported**	42
When a mistake is made, but is caught and corrected before affecting the patient, it is reported	46
When a mistake is made, but has no potential to harm the patient, it is reported	36
When a mistake is made that could harm the patient, but does not, it is reported	43
**D3: Supervisor/Manager expectations and actions promoting patient safety**	59
Manager says a good word when he/she sees a job done according to established patient safety procedures	73
Manager seriously considers staff suggestions for improving patient safety	65
Whenever pressure builds up, my manager wants us to work faster, even if it means taking shortcuts	48
My manager overlooks patient safety problems that happen over and over	51
**D4: Organizational learning and continuous improvement**	56
We are actively doing things to improve patient safety	69
Mistakes have led to positive changes here	56
After we make changes to improve patient safety, we evaluate their effectiveness	66
We are given feedback about changes put into place based on event reports	31
We are informed about errors that happen in the facility	54
In this facility, we discuss ways to prevent errors from happening again	61
**D5: Teamwork within units**	68
People support one another in this facility	61
When a lot of work needs to be done quickly, we work together as a team to get the work done	69
In facility, people treat each other with respect	78
When one area in this unit gets really busy, others help out	65
**D6: Communication openness**	52
Staff will freely speak up if they see something that may negatively affect patient care	60
Staff feel free to question the decisions or actions of those with more authority	43
Staff are afraid to ask questions when something does not seem right	52
**D7: Non-punitive response to error**	37
Staff feel like their mistakes are held against them	31
When an event is reported, it feels like the person is being written up, not the problem	38
We work in ‘crisis mode’ trying to do too much, too quickly	42
**D8: Staffing**	34
We have enough staff to handle the workload	21
Staff in this facility work longer hours than is best for patient care	37
We work in ‘crisis mode’ trying to do too much, too quickly	45
**D9: Management support for patient safety**	48
Management provides a work climate that promotes patient safety	49
The actions of management show that patient safety is a top priority	50
Management seems interested in patient safety only after an AE happens	34
Units work well together to provide the best care for patients	58
**D10: Teamwork across units**	40
There is good cooperation among units that need to work together	51
Units do not coordinate well with each other	42
It is often unpleasant to work with staff from other units	39
Things ‘fall between the cracks’ when transferring patients from one unit to another	32
Important patient care information is often lost during shift changes	42
Problems often occur in the exchange of information across units	35

## Discussion

The concept of patient safety in primary care has increased in their importance among the major international health organizations. Considering the majority of health care is delivered at the primary level, primary care is a critical area for patient safety research [20]. Recognizing the necessity of quantifying patient safety culture in primary care to improve patient safety, various studies have attempted to measure professionals PSC perception in this environment [21].

Hence the purpose of this investigation is to conveys an overall assessment of staff perceptions of safety culture in Moroccan primary care institutions [22–24]. It is the first of these kinds of studies to analyze the current condition of patient safety culture in primary care environment in Morocco.

The high rate of participation and response in this survey, with answers coming from a diverse group of frontline primary health care practitioners indicate the importance of patient safety issue in primary care. The dimension of “overall perception of safety” had a score of (56%). In addition, participants generally assessed patient safety in primary care acceptable or very good, with a fairly positive opinion of the level of patient safety. The dimension of “teamwork within units” received the highest score (69%), professionals communication within units has been found to be of excellent level in terms of cooperation in care and assisting co-workers, and the majority of staff interviewed perceives freedom of expression favorably which was comparable to previously published studies [25, 26]. The atmosphere reflected a strong feeling of organizational teamwork and systems to enable continual improvement at the unit level. The following might be because primary healthcare centers are small structures with less staff than hospitals and critical care units, and they are basic surroundings that encourage teamwork. The current research’s results indicate that various safety culture dimensions are viable areas for improvement. There are 5 safety dimensions with low positive responses (less than 50%) that should be prioritized. The lowest-scoring safety dimension is staffing (34%), non-punitive response to errors (37%), followed by team work across units (40%), frequency of event reporting (42%), management support for patient safety (48%). The dimension of “Staffing” ranking a very low score revealed that there are insufficient employees to meet the demand, in addition to extended working hours. Such circumstances create a state of urgency and disorganization, with employees working in ‘crisis mode,’ attempting to do too much, too quickly. In fact, a lack of staff, work overload, and an uncomfortable work atmosphere all have negative impacts on patients and Favor the likelihood of errors. Professionals stated that they did not have sufficient staff. to keep up with the amount of work, and that they worked longer hours than are optimal for patient care. This circumstance could have serious ramifications for patient safety and treatment quality [27]. They discovered that inadequate nurse staffing (fewer registered nurses), greater workload, and an unstable nursing environment were associated with unfavorable patient outcomes such as falls and prescription mistakes [28].

Furthermore, participants had a negative perception of the dimension “nonpunitive response to error” (37%). In fact, this dimension received the lowest score in many previous research [29] and is seen as a global issue [30]. According to the responses in our study, workers believe that when an error is made, it is the professional’s fault. It is the professional who is being blamed, not the problem. As a result of the ‘’blaming and shaming’’ culture in which failure is penalized or buried and individuals refuse to accept that issues exist. On the other hand, the dimension of “Frequency of adverse events reported” earned a low score of (42%) percent as well. This might be explained by the absence of a reporting culture in addition to the reality that errors are always viewed as a lack of competence and are rarely viewed as an opportunity for growth. The time it required to disclose and discuss an occurrence, fear of embarrassment, and vagueness of definition were all barriers to reporting within the practice [31]. Fear of being blamed, reputational and patient confidence damage, a lack of clarity about who to report to, and a lack of response were all barriers to external reporting. These results lead to the conclusion that the two dimensions “non-punitive response to error (37%)” and “frequency of event reporting (42%”)” seemed to be closely linked to each other. A low ranking for the dimensions “teamwork across units” (40%) and management support for patient safety (48%) reveals issues with team communication and teamwork management support. These issues are one of the foremost frequent causes of errors in primary care procedures. Indeed, unit contributions and teamwork are critical in order to provide continuity and quality of treatment for patients in the primary care centers. However, the shortage of a quality management structure for patient safety support in the majority of our institutions could partly explain the low perception of the previous two dimensions. A developed PSC and proper leadership are crucial steps in resolving these issues.

Our overall patient safety culture score finding, however, was lower than those reported from Greece (77%) [32], Egypt (68%) [33], Brazil (67%) [34] to Kuwait (57.5%) [35] of studies in primary health care facilities. Whereas differences in infrastructure and economics may explain some of these differences, the role of management and organizational dedication, leadership, and connections among health professionals should have held an essential part. In addition, the results obtained from primary care are comparable to those from a study carried with health care personnel in tertiary care facilities in Morocco (*n* = 204) [36]. In that study, the total positive response was 52%, and the dimensions with the lowest ratings were also staffing. This finding was consistent with the findings of AL Lawati et al. Oman (2019) [37], that various safety culture aspects are subjects for development. The weakest safety dimension is staffing (23%), followed by non-punitive response to errors (27%), frequency of event reporting (40%), and errors occurring when transferring patients to higher levels of health care during handoffs and transitions (46%). Consequently, to the similarity between these results and our findings, it is critical to promote a culture in which health workers are encouraged and supported to detect and report adverse events without fear of repercussions or blame. Reporting AEs is a critical component of good patient safety procedures, which involve error discovery, reporting, analysis, and corrective measures [38].

We advocate systematic strengthening of staff abilities through training and educational interventions that encourage an improved knowledge of teamwork principles, help individuals in acknowledging each other’s roles and perspectives, and create effective communication methods. Additionally, we suggest increased patient safety training for nurses at the levels of practice, policy, administration, research, and curriculum. Prioritizing risk analysis and management by training and increasing staff knowledge of the culture of safety and the AE report, obtaining resources from the institution’s health management team, and reviewing the success of teams in units [39]. Improving care quality and patient safety requires the implementation of a quality management system, as well as managerial training in communication and nursing leadership. Also reducing the sense of individualized of the error and blame culture by building shared responsibility for care and conducting a multifactorial and multidisciplinary studies.

Furthermore the engagement of administrative personnel in unit concerns and improved communication between administrators and caregivers in order to modify human resources in terms of number and availability [40] in addition to improving collaboration across units and professionals’ quality of life at work by developing a better communication system across units [41].

These strategies of communication and organization have several long-term benefits, including increased AE reporting and the incorporation of a more professional terms, team freedom to be included in a risk management strategy, increased feedback and analysis, and improved interaction between professionals across units [42].

### Strengths and limitations

To the best of our knowledge, the present study is one of the few studies that have dealt with the subject of the culture of healthcare safety and also the first to explore the subject in primary healthcare settings in Morocco. As a result, it lays out the foundation for similar studies in the future. The fact that the study was based on a questionnaire to assess the culture of care safety may inspire future research through focus groups and interviews, as well as practical observations and techniques in the same places of study for qualitative analysis. Also, this study allowed us to broaden the knowledge of quality of care, patient safety and the culture of care safety in primary healthcare.

The first limitation of this study is that it measures the perception of professionals with regard to the safety of care which depends largely on the knowledge of these professionals on the safety of care. Further, the instrument used in this study, although developed for the hospital setting, has been translated and validated in several contexts and widely used in primary healthcare settings. However, a similar instrument purposely developed to measure patient safety culture in primary care settings was recently translated and validated in the French context after the commencement of this study. Future researchers in Morocco can also adopt this new instrument to pave the way for comparing results across a variety of study instruments. Finally, the absence of studies concerning this subject on the national scale constituted a constraint preventing the discussion of our results with other national results.

## Conclusion

The importance of the safety culture for the safety of care lies in the fact that it drives the development of a coherent and integrated set of professional behaviors, thus improving the performance of healthcare organizations.

The culture of care safety tends towards a negative culture in this study with the dimensions of staffing, non-punitive response to error, frequency of reporting adverse events, teamwork across units and managements’ support for safety culture all had scores below 50%. These dimensions are to be developed as priorities, of course in addition to the rest of the dimensions, since this study did not reveal any dimension to be developed in the environment studied.

This study shed light on the state of care safety culture in primary health care facilities in Morocco. Based on the findings of this study, the authors recommend that all stakeholders; healthcare personnel, administrators, policy-makers, etc. need to make patient safety a top priority and instill a safety culture in their care environments to ensure safer care for patients.

### Electronic supplementary material

Below is the link to the electronic supplementary material.

Supplementary Material 1

## Data Availability

All the materials and data are available with the corresponding author for any necessary reviews.

## Abbreviations

AHRQ: Agency of Healthcare Research and Quality
HSOPSC: Hospital Survey on Patient Safety Culture
AE: Adverse events
PSC: Patient safety culture
CCCEQA: Coordination Committee of the Clinical Evaluation and Quality in Aquitaine
